# Causal relationship between gastroesophageal reflux disease, Barrett's esophagus, and epilepsy: A bidirectional Mendelian randomization study

**DOI:** 10.1002/brb3.3117

**Published:** 2023-06-08

**Authors:** Xiaoduo Liu, Tao Wei, Lubo Shi, Shaojiong Zhou, Yufei Liu, Weiyi Song, Xinwei Que, Zhibin Wang, Yi Tang

**Affiliations:** ^1^ Department of Neurology & Innovation Center for Neurological Disorders Xuanwu Hospital, Capital Medical University, National Center for Neurological Disorders Beijing China; ^2^ Department of Gastroenterology, Beijing Friendship Hospital, Capital Medical University National Clinical Research Center for Digestive Diseases Beijing Digestive Disease Center Beijing China; ^3^ Neurodegenerative Laboratory of Ministry of Education of the Peoples Republic of China Beijing China

**Keywords:** Barrett's esophagus, epilepsy, gastroesophageal reflux disease, mendelian randomization

## Abstract

**Background:**

The incidence of gastroesophageal reflux disease (GERD) has been shown to be elevated in individuals with epilepsy. Traditional observational studies have led to a limited understanding of the effects of GERD and BE on epilepsy due to the interference of reverse causation and potential confounders.

**Methods:**

We conducted a bidirectional two‐sample Mendelian randomization (MR) analysis to determine whether GERD and BE can increase the risk of epilepsy. Genome‐wide association study data on epilepsy and its subgroups were obtained from the International League Against Epilepsy consortium for primary analysis using three MR approaches and the FinnGen consortium for replication and meta‑analysis. We calculated causal estimates between the two esophageal diseases and epilepsy using the inverse‐variance weighted method. Sensitivity analysis was conducted to detect heterogeneity and pleiotropy.

**Results:**

We found a potential effect of genetically predicted GERD on the risk of epilepsy (odds ratio [OR] = 1.078; 95% confidence interval [CI], 1.014–1.146, *p* = .016). Specifically, GERD showed an effect on the risk of generalized epilepsy (OR = 1.163; 95% CI, 1.048–1.290, *p* = .004) but not focal epilepsy (OR = 1.059, 95% CI, 0.992–1.131, *p* = .084). Notably, BE did not show a significant causal relationship with the risks of generalized and focal epilepsy.

**Conclusions:**

Under MR assumptions, our findings suggest a potential risk‐increasing effect of GERD on epilepsy, especially generalized epilepsy. Considering the exploratory nature of our study, the association between GERD and epilepsy needs to be confirmed by future prospective studies.

## Graphical abstract

Genetically predicted GERD leads to a potential increased risk of epilepsy, especially generalized epilepsy. Genetically predicted BE and epilepsy are found to have no causal relationship. Neurostimulation may be a possible mechanism that mediates GERD leading to epilepsy.

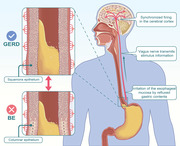

## 1 INTRODUCTION

Epilepsy is clinically characterized by two seizures occurring greater than 24 h apart or diagnosis of an epilepsy syndrome, and more than 70 million people are affected globally (Fisher et al., 2014; Thijs et al., 2019). A meta‐analysis suggested a weighted median standardized mortality ratio of 2.3 for patients with epilepsy of all ages relative to the general population, and higher in children (Thurman et al., 2017). The premature death of population with epilepsy is a major public health problem, and early screening and prevention of epilepsy is a high priority (de Boer et al., 2008). Gastroesophageal reflux disease (GERD), a common digestive tract disease, is described as symptoms of heartburn and reflux caused when acidic stomach contents enter the esophagus (Maret‐Ouda et al., 2020). A previous study showed a statistical association between psychogenic non‐epileptic seizures and GERD in Asian Americans but did not find an association between GERD and epilepsy after multivariate analysis (Gorenflo et al., 2022). However, the causal relationship between GERD and epilepsy remains unclear. Investigating the causal relationship of GERD with epilepsy potentially sheds light on managing epilepsy risk and reducing its recurrence in patients.

The literature on the association between GERD and epilepsy is limited. A potential correlation has been demonstrated between brain electrical activity and gastric acid‐related stimulation in which paraesophageal sensory nerves mediate esophageal sensation in response to acid stimulation, leading to alterations in electroencephalogram (EEG) results (Kim et al., 2017). The activation of multiple brain regions recorded by functional magnetic resonance imaging and increased functional connectivity reflect the potential connection between GERD and epilepsy (Kern et al., 2004; Ribeiro et al., 2022). Further, GERD may be associated with epilepsy, and the two often occur concomitantly (Byard, 2006; Fiorentino et al., 2009; Nishiyama et al., 2020). Irritation of the esophageal mucosa by stomach contents can lead to chronic esophageal injury and further induce chronic inflammation of the esophageal mucosa and cause the production of columnar epithelial metaplasia with cancerous tendencies (termed Barrett's esophagus [BE]) (Spechler & Souza, 2014). As a common complication of GERD, BE reflects the result of long‐term gastric acid‐related irritation and is accompanied by damage to afferent nerves (Woodland et al., 2013).

Due to design flaws in conventional studies, existing observational studies often fail to fully exclude reverse causality and confounding factors (Sekula et al., 2016). Mendelian randomization (MR) analysis is used as a genetic epidemiology method, which involves the use of genetic variants as tool variables to explore causal relationships between exposures (e.g., GERD and BE) and outcomes (e.g., epilepsy) (Emdin et al., 2017). By using genetic alleles randomly assigned to offspring before birth, MR has shown advantages in excluding confounding factors and identifying the causal determinants of outcomes (Davey Smith & Hemani, 2014). In this study, we applied MR analysis to explore the possible effects of GERD and BE on epilepsy. Furthermore, we investigated whether GERD and BE have differential effects on generalized and focal epilepsy. The effect of epilepsy on GERD and BE was also investigated by reverse MR analysis.

## 2 MATERIALS AND METHODS

### 2.1 Study design

**FIGURE 1:**
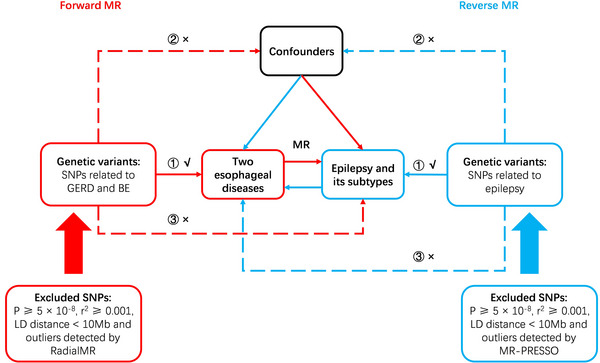
Flowchart of bidirectional Mendelian randomization analysis. BE, Barrett's esophagus; GERD, gastroesophageal reflux disease; IVW, inverse‐variance weighted; SNP, single nucleotide polymorphism; MR‐PRESSO, Pleiotropy Residual Sum and Outlier.

We conducted a univariable bidirectional two‐sample MR analysis to explore the causal relationship of GERD and BE with the risk of epilepsy. Genome‐wide association study (GWAS) data for epilepsy from two independent consortiums were used for replication and meta‐analysis. The overall design of our MR analyses can be found in Figure 1. To explore causal effects using MR methods, our genetic instrumental variants (IVs) should satisfy three assumptions: (1) the genetic IVs we extracted should be strongly related to GERD and BE (*P* < 5 × 10^−8^), (2) the genetic IVs were not allowed to be associated with any confounding factors that could lead to pleiotropy, and (3) the effect of the genetic IVs on the outcomes could only be mediated by the exposures (Emdin et al., 2017). This study used publicly available GWAS pooled data, so ethical approval was not required.

### 2.2 Data sources

We summarized GWAS data on GERD, BE, and epilepsy from available published studies (Table S1). The GWAS data of GERD and BE used in our MR analysis were from a recent pooled GWAS (accessed via study accession GCST90000514 and GCST90000515), including GERD (129,080 cases and 473,524 controls) and BE (13,358 cases and 43,071 controls) (Ong et al., 2022). GWAS data on epilepsy, generalized epilepsy, and focal epilepsy were extracted from the International League Against Epilepsy (ILAE). Summary GWAS data for epilepsy and its subgroups (including generalized and focal epilepsy) were used to extract suitable genetic IVs. There were 15,212 epilepsy cases, 3769 generalized epilepsy cases, 9671 focal epilepsy cases, and 29,677 controls (nearly 86% were European) (International League Against Epilepsy Consortium on Complex Epilepsies 2018). All these publicly available data can be accessed on the website (https://gwas.mrcieu.ac.uk).

To ensure the stability of significant results, we extracted GWAS data on epilepsy (10,354 cases and 264,662 controls), generalized epilepsy (1160 cases and 332,143 controls), and focal epilepsy (5922 cases and 332,145 controls) from the FinnGen Consortium Freeze 8 database for replication and meta‐analysis (J. Cai et al., 2022).

### 2.3 Instrument selection

The single nucleotide polymorphisms (SNPs) were extracted as IVs with a genome‐wide strict significance threshold (*p* < 5 × 10^−8^). Moreover, we removed the SNPs that were in linkage disequilibrium (LD; *r* ^2^ < 0.001) within a 10‐Mb window, and the remaining SNPs were retained and extracted (Shi et al., 2022). If the SNPs that represented the outcome were not available in GWAS data, we used proxy SNPs in LD (*r* ^2^ > 0.8) that could be found to replace them (Zhuang et al., 2020). Then, we performed harmonization, and the SNPs that were palindromic with an intermediate allele frequency were removed. After the removal of SNPs strongly associated with the outcome, we performed a bidirectional MR analysis. Furthermore, we calculated the *F* statistic to ensure that there was no weak IV bias, and an *F* statistic >10 was considered robust enough to counter weak instrument bias (Brion et al., 2013). The *R* ^2^ and F statistic of each SNP were calculated according to the following formulas: *R* ^2^ = 2 × EAF × (1‐EAF) × *β* ^2^ and *F* statistic = *R* ^2^ × (*N* – 2)/(1 – *R* ^2^) (Shi et al., 2022). Moreover, we used the PhenoScanner database (Version 2, http://www. phenoscanner.medschl.cam.ac.uk/) to detect other genome‐wide significant (*P* < 5 × 10^−8^) traits associated with the genetic variants that may act as potential exposure factors (Staley et al., 2016).

### 2.4 Primary analysis

We used three MR approaches to determine MR estimates of the effect of esophageal disease on epilepsy, including the inverse‐variance weighted (IVW), weighted median (WM), and MR‒Egger methods. Multiple methods were used, as they had different underlying assumptions for horizontal pleiotropy. The IVW method summarized the weighted average of Wald ratio estimates of the causal effects for each variant, and it had the highest statistical efficiency among the methods (Burgess et al., 2013). All extracted SNPs were considered effective by the IVW method, which was regarded as the main result of our research when no horizontal pleiotropy existed (Y. Cai et al., 2021). The assumption of the MR Egger test was that all genetic variations would have different pleiotropic patterns from the exposure. When all genetic variants were invalid IVs, the MR‒Egger test yielded a valid test of the null causal hypothesis and a consistent causal estimate despite this method exhibiting low statistical precision and being susceptible to outlying genetic variants (Bowden et al., 2015). The WM method assumed that at least 50% of the weight of the valid IVs was needed. It was significantly and consistently more precise than the MR‒Egger method and more robust to violations of causal effects (Bowden et al., 2016).

### 2.5 Sensitivity analysis

We further performed various sensitivity analyses to assess the robustness of the results. Cochran's *Q* statistic was used to assess heterogeneity if the *p*‐value was <.05, which revealed that the results might have heterogeneity. Forest plots and leave‐one‐out analysis were used to observe whether there was a single SNP causing bias in the results. The MR‒Egger intercept and Mendelian Randomization Pleiotropy Residual Sum and Outlier (MR‐PRESSO) tests were used to detect horizontal pleiotropy. A *p*‐value <.05 indicated that the results might be invalid due to horizontal pleiotropy. RadialMR has a more direct effect on detecting outliers (Bowden et al., 2018). When horizontal pleiotropy persisted (global *p* <.05) after the application of MR‐PRESSO, the radial MR method was applied to detect and remove outliers. The MR analysis was performed again after the outliers were removed.

We used the Benjamini‒Hochberg method to calculate a false discovery rate (FDR)‐corrected *p*‐value to adjust the results of the MR analysis (Shi et al., 2022). A *p*‐value less than the *P*_FDR_ was considered to indicate a significant correlation, while a *p* <.05 but greater than the *P*_FDR_ was considered to indicate a suggestive association.

### 2.6 Replication and meta‐analysis

To validate the robustness of the results, the FinnGen GWAS database was used as another independent consortium for epilepsy data. We performed a replicate MR analysis for significant results and a meta‐analysis to explore the combined effect. All statistical analyses were performed by using the “TwoSampleMR” (Hemani et al., 2018), “MR‐PRESSO” (Verbanck et al., 2018) and “RadialMR” packages (Zhang et al., 2021) in R (version 4.2.1.) and Reviewer Manager software (Version 5.4.1).

## 3 RESULTS

For esophageal diseases, we obtained 74 SNPs associated with GERD and 15 SNPs associated with BE (Table S2‐S3). Moreover, for epilepsy, we found three SNPs associated with epilepsy, nine associated with generalized epilepsy, and one associated with focal epilepsy (Table S4). *F* statistics reflected a strong correlation between the IVs and the exposure (*F* ranged from 37.861 to 669.242). We did not find other SNPs that might act as potential exposure factors affecting epilepsy by searching the PhenoScanner database (Verbanck et al., 2018).

### 3.1 Primary analysis

**FIGURE 2:**
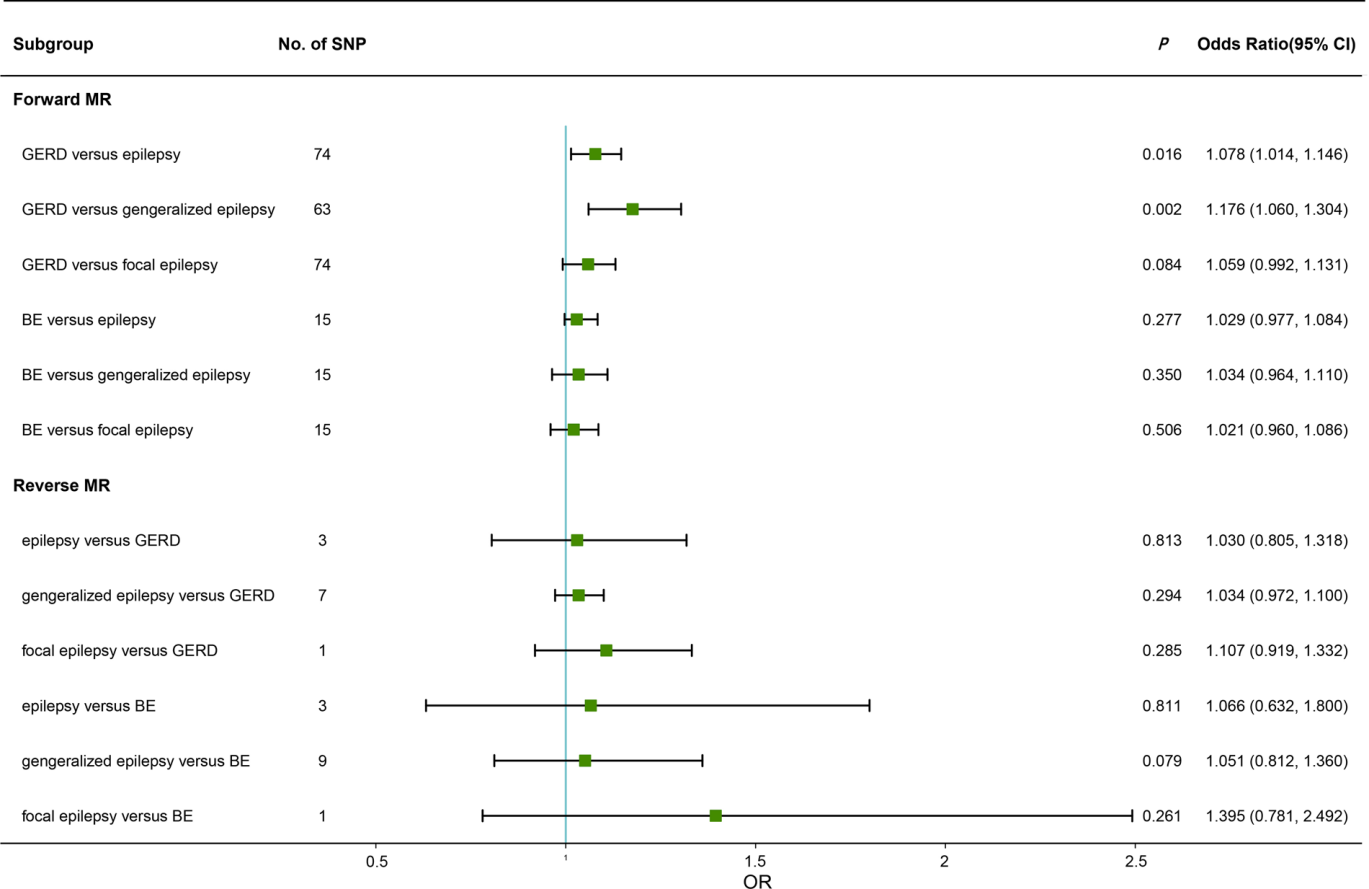
Estimation of two‐sample Mendelian randomization analysis for the two esophageal diseases and epilepsy. BE, Barrett's esophagus; CI, confidence interval; GERD, gastroesophageal reflux disease; MR, Mendelian randomization; OR, odds ratio; SNP, single nucleotide polymorphism.

The final estimated value of the causal effect by the IVW is presented in Figure 2. Together with MR‐PRESSO, MR Egger, and WM methods, all results are shown in Tables 1 and 2. In the forward MR analysis, genetic predispositions to GERD were related to epilepsy (odds ratio [OR] = 1.078, 95% confidence interval [CI], 1.014–1.146, *p* = .016, *P*_FDR_ = 0.096) and generalized epilepsy (OR = 1.176, 95% CI, 1.060–1.304, *p* = .002, *P*_FDR_ = 0.024) under the IVW model, while GERD had no causal relationship with focal epilepsy (OR = 1.059, 95% CI, 0.992–1.131, *p* = .084, *P*_FDR_ = 0.252). The causal implication between GERD and epilepsy should be treated with caution due to FDR correction. We also found no causal relationship between BE and epilepsy (OR = 1.029, 95% CI, 0.977–1.084, *p* = .277, *P*_FDR_ = 0.441), generalized epilepsy (OR: 1.034, 95% CI, 0.964–1.110, *p* = .350 *P*_FDR_ = 0.467), or focal epilepsy (OR = 1.021, 95% CI, 0.960–1.086, *p* = .506, *P*_FDR_ = 0.607).

**TABLE 1:** Forward Mendelian randomization (MR) results of two esophageal diseases and epilepsy.

Exposure	nSNPs	Method	OR (95% CI)	*p*‐Value	*Q* pval	Intercept *p‐*value	Global *P*	*P*_FDR_
GERD vs. epilepsy	74	IVW	1.078 (1.014, 1.146)	**.016**	0.127			0.096
		MR Egger	0.778 (0.538, 1.125)	.186		0.083		
		MR‐PRESSO	1.078 (1014., 1.146)	**.018**			0.129	
		WM	1.057 (0.972, 1.149)	.198				
BE vs. epilepsy	15	IVW	1.029 (0.977, 1.084)	.277	0.069			0.441
		MR Egger	0.833 (0.946, 1.702)	.833		0.891		
		MR‐PRESSO	1.029 (0.977, 1.083)	.295			0.074	
		WM	1.007 (0.946, 1.072)	.716				
GERD vs. generalized epilepsy	63	IVW	1.176 (1.060, 1.304)	**.002**	0.513			**0.024**
		MR Egger	0.846 (0.458, 1.563)	.721		0.290		
		MR‐PRESSO	1.176 (1.062, 1.302)	**.003**			0.524	
		WM	1.147 (0.990, 1.330)	.085				
BE vs. generalized epilepsy	15	IVW	1.034 (0.964, 1.110)	.350	0.407			0.467
		MR Egger	1.228 (0.671, 2.247)	.517		0.584		
		MR‐PRESSO	1.034 (0.999, 1.071)	.366			0.403	
		WM	1.061 (0.960, 1.110)	.246				
GERD vs. focal epilepsy	74	IVW	1.059 (0.992, 1.131)	.084	0.701			0.252
		MR Egger	0.804 (0.539, 1.200)	.289		0.175		
		MR‐PRESSO	1.059 (0.995, 1.127)	.073			0.704	
		WM	1.022 (0.930, 1.122)	.653				
BE vs. focal epilepsy	15	IVW	1.021 (0.960, 1.086)	.506	**0.049**			0.607
		MR Egger	1.003 (0.586, 1.716)	.992		0.947		
		MR‐PRESSO	1.021 (0.960, 1.087)	.517			0.053	
		WM	1.036 (0.962, 1.116)	.343				

Abbreviations: *P*‐value < 0.05 is set as nominal significant, whereas *P*_FDR_ < 0.05 is set as significant. BE, Barrett's esophageal disease; CI, confidence interval; GERD, gastroesophageal reflux disease; IVW, inverse‐variance weighted; MR‐PRESSO, Pleiotropy Residual Sum and Outlier; nSNPs, number of single nucleotide polymorphisms; OR, odds ratio; Q_pval, *p*‐value of the Cochran Q statistic; WM, weighted median.

**TABLE 2:** Reverse Mendelian randomization (MR) results of two esophageal diseases and epilepsy.

Exposure	nSNPs	Method	OR (95%CI)	*p*‐Value	Q pval	Intercept *p‐*value	Global *P*	*P*_FDR_
Epilepsy vs. GERD	3	IVW	1.030 (0.805, 1.318)	.813	**0.011**			0.813
		MR Egger	0.589 (0.306, 1.132)	.358		0.332		
		WM	1.093 (0.936, 1.277)	.260				
Generalized epilepsy vs. GERD	7	IVW	1.034 (0.972, 1.100)	.294	0.138			0.441
		MR Egger	1.006 (0.327, 3.096)	.993		0.963		
		MR‐PRESSO	1.034 (0.972, 1.100)	.334			0.173	
		WM	1.039 (0.971, 1.112)	0.266				
Focal epilepsy vs. GERD	1	Wald ratio	1.107 (0.919, 1.332)	.285				0.441
Epilepsy vs. BE	3	IVW	1.066 (0.632, 1.800)	.811	0.126			0.813
		MR Egger	0.422 (0.054, 3.311)	.562		0.528		
		WM	1.338 (0.842, 2.126)	.218				
Generalized epilepsy vs. BE	9	IVW	1.051 (0.812, 1.360)	.079	0.240			0.252
		MR Egger	5.044 (0.978, 26.000)	.094		0.119		
		MR‐PRESSO	1.147 (0.985, 1.337)	.117			0.263	
		WM	1.063 (0.885, 1.277)	.510				
Focal epilepsy vs. BE	1	Wald ratio	1.395 (0.781, 2.492)	.261				0.441

Abbreviations: *P*‐value < 0.05 is set as nominal significant, whereas *P*_FDR_ < 0.05 is set as significant. BE, Barrett's esophageal disease; CI, confidence interval; GERD, gastroesophageal reflux disease; IVW, inverse‐variance weighted; MR‐PRESSO, Pleiotropy Residual Sum and Outlier; nSNPs, number of single nucleotide polymorphisms; OR, odds ratio; Q_pval, *p*‐value of the Cochran Q statistic; WM, weighted median.

The results of several methods of reverse MR analysis showed that the genetically predicted risk of GERD and BE was not related to epilepsy and its subtypes (epilepsy vs. GERD: OR, 1.030, 95% CI, 0.805–1.318, *p* = .813, *P*_FDR_ = 0.813; generalized epilepsy vs. GERD: OR, 1.034, 95% CI, 0.972–1.100, *p* = .294, *P*_FDR_ = 0.441; focal epilepsy vs. GERD: OR, 1.107, 95% CI, 0.919–1.332, *p* = .285, *P*_FDR_ = 0.441; epilepsy vs. BE: OR, 1.066, 95% CI, 0.623–1.800, *p* = .811, *P*_FDR_ = 0.813; generalized epilepsy vs. BE: OR, 1.051, 95% CI, 0.812–1.360, *p* = .079, *P*_FDR_ = 0.252; focal epilepsy vs. BE: OR, 1.395, 95% CI, 0.781–2.492, *p* = .261, *P*_FDR_ = 0.441, Table 2). Moreover, we also present the raw results of the MR analysis before the outliers were removed (Tables S5 and S6).

### 3.2 Sensitivity analysis

In the sensitivity analysis of the forward MR analysis, we observed heterogeneity (*P_Q_* = 0.049) for the effect of BE on focal epilepsy. Heterogeneity is acceptable in this study since we used the random‐effects IVW as the primary result (Burgess et al., 2019). Other results did not show this phenomenon (GERD vs. epilepsy: *P_Q_* = 0.127; BE vs. epilepsy: *P_Q_* = 0.069; GERD vs. generalized epilepsy: *P_Q_* = 0.513; BE vs. generalized epilepsy: *P_Q_* = 0.407; GERD vs. focal epilepsy: *P_Q_* = 0.701). None of the MR Egger intercept test or MR PRESSO results showed the generation of horizontal pleiotropy (all *P* > 0.05). Although there was still heterogeneity after removing outliers by the MR‐PRESSO method, we used the RadialMR method to remove 11 outliers (Figure S9), making the results more reliable. Scatter and funnel plots also showed that the results tended to be stable (Figures S1–S6).

In the reverse analysis, Cochran's *Q* test revealed that there were heterogeneous effects of epilepsy on GERD (*P_Q_* = 0.011). However, no significant heterogeneity was observed in the other results (generalized epilepsy vs. GERD: *P_Q_* = 0.138, epilepsy vs. BE: *P_Q_* = 0.126; generalized epilepsy vs. BE: *P_Q_* = 0.240). The MR Egger intercept test and MR‐PRESSO proved that the results did not have horizontal pleiotropy. It was remarkable that at first, there was a clear horizontal pleiotropic pattern of this result for the effect of generalized epilepsy on GERD (Table S3). After correcting for two outliers (rs2833098 and rs68082256), the global *P* of MR‐PRESSO became insignificant. *F* statistics >10 showed the attributes of extracted IVs that were strongly correlated with intermediate phenotypes (Tables S2–S4). The forest plots and leave‐one‐out analysis showed no significant bias for an SNP (Figures S7–S8).

### 3.3 Replication and meta‑analysis

**FIGURE 3:**
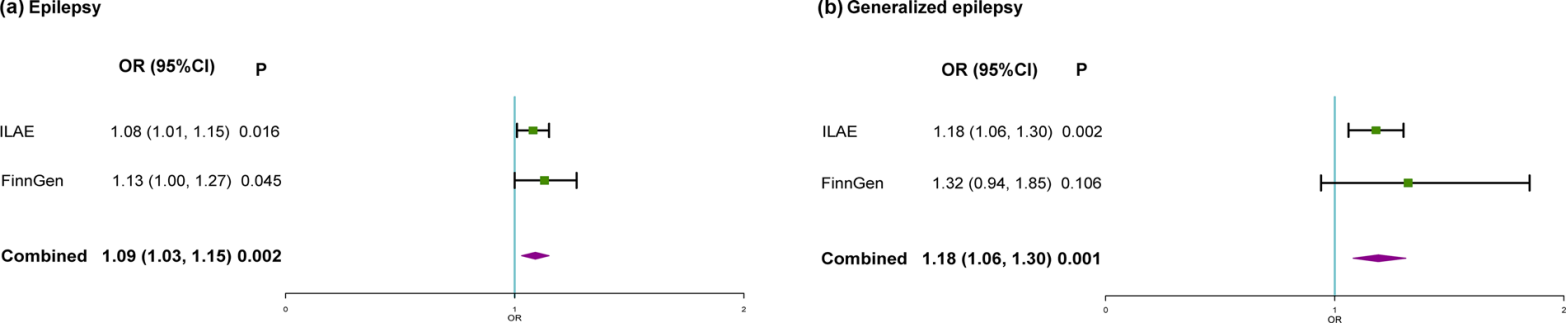
A meta‐analysis of the causal association of GERD with epilepsy and generalized epilepsy. CI, confidence interval; GERD, gastroesophageal reflux disease; ILAE, the International League Against Epilepsy; OR, odds ratio.

To verify the stability of the results, another independent FinnGen database was used for repeated MR analysis of the results, and further meta‐analysis was performed (Figure 3). MR analysis of GERD showed a similar trend in the FinnGen consortium and remained significant in the combined analysis of ILAE and FinnGen data (epilepsy: OR, 1.09, 95% CI, 1.03–1.15, *p* = .002; generalized epilepsy: OR, 1.19, 95% CI, 1.08–1.31, *p* = .001).

## 4 DISCUSSION

**FIGURE 4:**
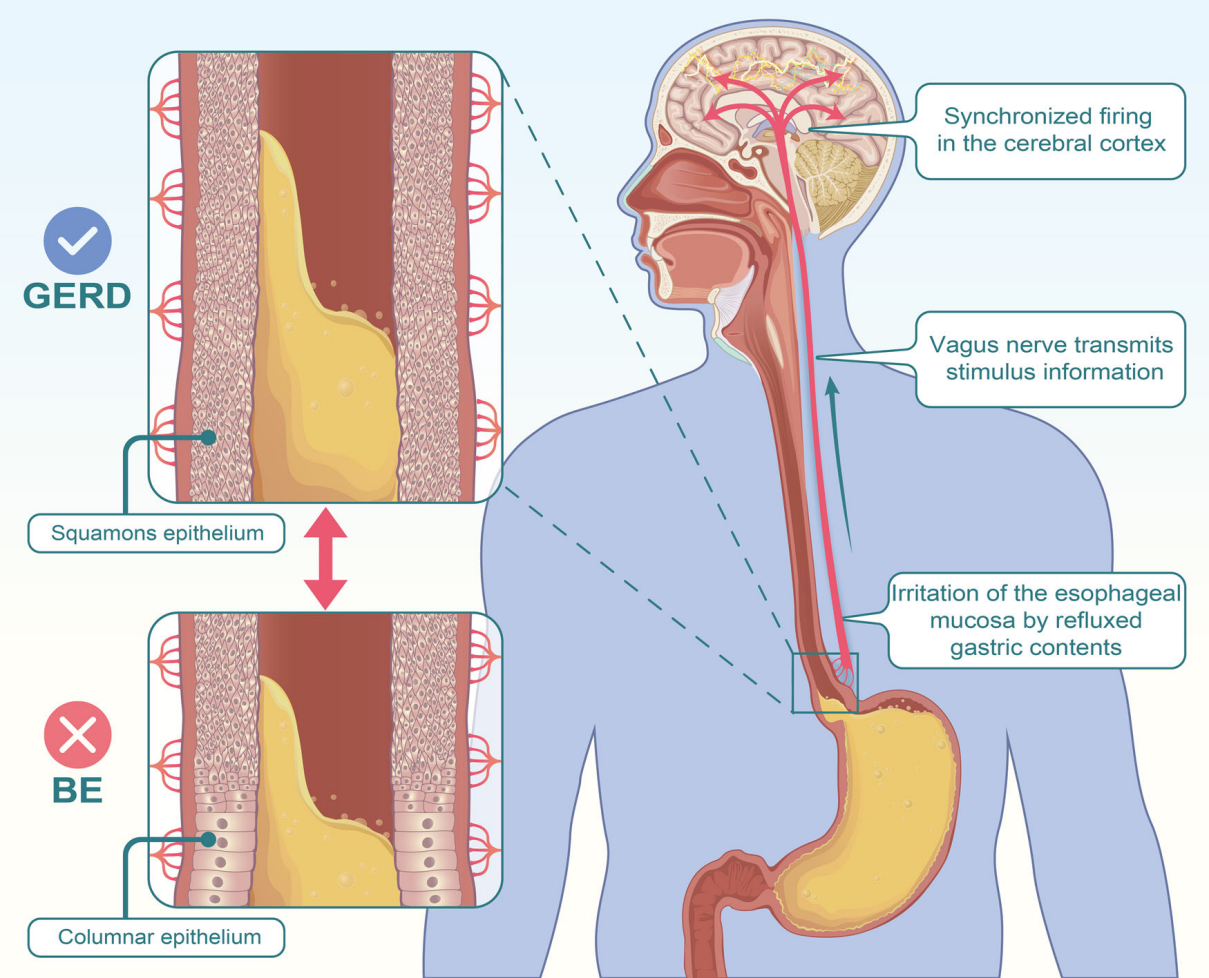
Possible mechanism of GERD as a trigger leading to epilepsy. Refluxed stomach acid can activate the autonomic nervous system, further leading to altered excitability of the whole brain. However, BE fails to generate the corresponding outcomes due to the columnar epithelial metaplasia. BE, Barrett's esophagus; GERD, gastroesophageal reflux disease.

The study suggests that the genetic liability of GERD plays a potential role in the increased risk of epilepsy. However, considering that our significant or nominally significant OR is close to 1, our results are suggestive and need to be treated with caution. Specifically, the genetic prediction of GERD suggests a latent impact on the risk of generalized epilepsy rather than focal epilepsy. This result suggested possible mechanisms of neurostimulation in a wide range of brain areas due to stimulation of gastric contents to vagus nerve (Figure 4). Notably, BE had no causal relationship with epilepsy and its subtypes. Further, we found that epilepsy had no prominent risk‐increasing effect on these two esophageal diseases. Considering the likely effect of GERD on developing epilepsy, our study provides an insight into reducing the risk of seizure recurrence in patients by emphasizing monitoring and improving their esophageal condition.

Our study provides evidence for the genetic prediction of GERD as a potential risk factor for epilepsy (particularly generalized epilepsy). As one of the main information afferent pathways connecting the digestive tract to the brain, the complex autonomic nervous system transmits information received by nerve endings of the esophageal mucosa from gastric acid‐related stimulation to the nucleus of the solitary tract of the brainstem (Yu et al., 2021), where afferent vagal projections are transmitted through the pontine parabrachial nucleus and thalamus to seizure‐generating regions in the basal forebrain and insular cortex (Ohemeng & Parham, 2020). It is noteworthy that previous observational studies did not find a statistically significant relationship between GERD and epilepsy (Gorenflo et al., 2022). We are inclined to the view that GERD‐induced neurostimulation acts as a trigger for abnormal synchronous discharges of the cerebral cortex in some patients. Ethnicity as a confounding factor may contribute to the results.

Our results show that genetically predicted GERD is a potential risk factor for generalized epilepsy but not for focal epilepsy. Generalized epilepsy involves the broader epileptogenic network, especially the bilateral thalamocortical structures, while the focal epilepsy network involves neuronal circuits in only one hemisphere (Thijs et al., 2019). Thalamocortical structures, as the primary mechanism of vagal mediation of esophageal pain sensation, exert a broad influence between cortical networks and have extensive connectivity with the wide cerebral cortex rather than being confined to a certain part of the brain region (Buckner & DiNicola, 2019; Groh et al., 2018). This is consistent with previous findings that patients with GERD have a wider range of activated brain areas (including the sensory/motor, parieto‐occipital, cingulate, prefrontal, insular, and anterior cingulate cortices) (Kern et al., 2004). Different parameters of neural stimulation determine the outcome of cortical synchronization or desynchronization (Chase et al., 1966; Cukiert et al., 2013). Vagus nerve stimulation through specific rhythm settings, as an important treatment for intractable epilepsy, is more effective for generalized epilepsy than focal epilepsy, which further supports the global effect of vagus nerve stimulation on multiple brain regions (Englot et al., 2011; Montavont et al., 2007).

Notably, BE and epilepsy did not show a causal relationship in this study, although very limited cases suggested a history of BE in epileptic patients (Delgado et al., 2013; Finsterer, 2021). Since BE is characterized by a lower neurosensitivity to painful stimuli from gastric acid, patients with BE do not show significant effects of GERD in terms of vagal activation due to gastric acid‐related stimulation (Weijenborg et al., 2017). Furthermore, the mucous membrane of the esophagus, with its metaplasia of the columnar epithelium, acts as an adaptive change in response to stimulation by gastric contents and reduces the irritating effect of gastric acid (Dvorak et al., 2009). Apart from this, the GWAS sample size for the BE phenotype is relatively small, and further studies including larger populations are needed to assess the effect of BE on the development of epilepsy. Collectively, our results suggest that BE does not have a causal effect on developing epilepsy.

Understanding the potential association between GERD and epilepsy by our MR study may help to reduce the likelihood of recurrence in population with epilepsy as well as to prevent and screen for epilepsy early through the prevention or treatment of GERD. Treatment of GERD with fundoplication has demonstrated control of seizure symptoms (AlNamshan et al., 2019; Fiorentino et al., 2009). As another common treatment for GERD, gastrostomy reduces the rate of seizure hospitalization for patients by 20% after 5 years (Jacoby et al., 2020). In addition, clinicians should increase the application of EEGs and neuroimaging in patients with GERD; patients with epilepsy should be recommended on routine examination, such as gastrointestinal endoscopy, to detect the presence of GERD as a comorbidity (Thijs et al., 2019). Considering the possible triggering effect of neurostimulation in the process of GERD leading to epilepsy, gastric vagotomy, moderate fasting, and laryngeal nerve stimulation may be effective methods to reduce acid reflux and prevent epilepsy (Budde et al., 2020). More prospective studies are required to further explore the causal relationship between GERD and epilepsy. It has to be emphasized again that our weak effect values are only clinically suggestive rather than conclusive. Thus, the exploration and implementation of clinical significance need to be kept with caution.

Several limitations in our study warrant caution. First, studies have uncovered an inverse association of gastroesophageal reflux symptoms with epilepsy by affecting the function of the lower esophageal sphincter or congenital genetic abnormalities, which could not be confirmed in our analysis (Harrington et al., 2004; Kim et al., 2017). However, our study did not reveal a similar effect of epilepsy on GERD. Second, the risk of epilepsy may be time dependent, and we should remain informed about clinical interventions related to these two diseases. Third, the GWAS summary datasets used in this study are limited to individuals of European origin, and the possibility of residual confounding from other variables cannot be completely ruled out. It is unclear whether we can extrapolate our findings to general populations. Fourth, we lack more finely stratified GWAS data for further subgroup analysis due to the overlap of patients with BE and GERD. Possible misdiagnosis between non‐erosive reflux disease and epilepsy is also a likely cause of the outcome.

## 5 CONCLUSION

Our study explains the genetic prediction of GERD leading to an increased potential risk of epilepsy, particularly generalized epilepsy. Yet, our study is only exploratory. Future studies on these two esophageal disorders will help to explore the mechanisms of epilepsy development and reduce the recurrence of epilepsy.

## AUTHOR CONTRIBUTIONS

Yi Tang and Zhibin Wang conceived the hypothesis. Xiaoduo Liu, Tao Wei, and Lubo Shi designed the study, analyzed the data, and drafted the manuscript. Xiaoduo Liu, Tao Wei, Shaojiong Zhou, and Yufei Liu interpreted the analysis results. Xiaoduo Liu, Tao Wei, Weiyi Song, and Xinwei Que were responsible for data management. Yi Tang and Zhibin Wang reviewed and revised the manuscript. All authors approved the final version of the manuscript.

## CONFLICT OF INTEREST STATEMENT

The authors declare no conflict of interest.

### PEER REVIEW

The peer review history for this article is available at https://publons.com/publon/10.1002/brb3.3117.

## Supporting information


**Table S1**. Detailed information of the studies and datasets used for MR analysis
**Table S2**. Characteristics of selected SNPs associated with gastroesophageal reflux disease
**Table S3**. Characteristics of selected SNPs associated with Barrett's esophagus.
**Table S4**. Characteristics of selected SNPs associated with epilepsy and its subtypes.
**Table S5**. Forward causal relationships of two esophageal disease with epilepsy performed by MR before removing the outliers.
**Table S6**. Reverse causal relationships of epilepsy with two esophageal disease performed by MR before removing the outliers.
**Figure S1**. The causal effect of GRED on epilepsy risk. (A) Scatter plot, (B) Funnel plot, (C) Forest plot, and (D) Leave one out plot. GERD, gastroesophageal reflux disease.
**Figure S2**. The causal effect of GRED on generalized epilepsy risk. (A) Scatter plot, (B) Funnel plot, (C) Forest plot, and (D) Leave one out plot. GERD, gastroesophageal reflux disease.
**Figure S3**. The causal effect of GRED on focal epilepsy risk. (A) Scatter plot, (B) Funnel plot, (C) Forest plot, and (D) Leave one out plot. GERD, gastroesophageal reflux disease.
**Figure S4**. The causal effect of BE on epilepsy risk. (A) Scatter plot, (B) Funnel plot, (C) Forest plot, and (D) Leave one out plot. BE, Barrett's esophagus.
**Figure S5**. The causal effect of BE on generalized epilepsy risk. (A) Scatter plot, (B) Funnel plot, (C) Forest plot, and (D) Leave one out plot. BE, Barrett's esophagus.
**Figure S6**. The causal effect of BE on focal epilepsy risk. (A) Scatter plot, (B) Funnel plot, (C) Forest plot, and (D) Leave one out plot. BE, Barrett's esophagus.
**Figure S7**. The causal effect of generalized epilepsy on GRED. (A) Scatter plot, (B) Funnel plot, (C) Forest plot, and (D) Leave one out plot. GERD, gastroesophageal reflux disease.
**Figure S8**. The causal effect of generalized epilepsy on BE. (A) Scatter plot, (B) Funnel plot, (C) Forest plot, and (D) Leave one out plot. BE, Barrett's esophagus.
**Figure S9**. Outliers identified by MR Radial.
