# Supplementary material for: Causal relationship between gastroesophageal reflux disease, Barrett's esophagus, and epilepsy: A bidirectional Mendelian randomization study
Source: Brain Behav. 2023 Jun 8;13(9):e3117. doi: 10.1002/brb3.3117 (PMC10498072; doi:10.1002/brb3.3117)

**Supplementary files**

**Supplementary Tables**

Table S1. Detailed information of the studies and datasets used for Mendelian randomization analysis……………………………………………………………………………2

Table S2. Characteristics of selected SNPs associated with gastroesophageal reflux disease…………………………………………………………………….….………………3

Table S3. Characteristics of selected SNPs associated with Barrett’s esophagus.……………………………………………………………………………….…...………………6

Table S4. Characteristics of selected SNPs associated with epilepsy……………………………………………………………………………………………...…………………7

Table S5. Forward causal relationships of two esophageal disease with epilepsy performed by MR before removing the outliers…………………….............………….………8

Table S6. Reverse causal relationships of epilepsy with two esophageal diseases performed by MR before removing the outliers …………………………….…………………9

**Supplementary Figures**

Figure S1. The causal effect of GRED on epilepsy risk………………………………………………………………………………………………………….…………………10

Figure S2. The causal effect of GRED on generalized epilepsy risk……………………………………………………………………………………………..…………………11

Figure S3. The causal effect of GRED on focal epilepsy risk……………………………………………………………………………………...………………..………………12

Figure S4. The causal effect of BE on epilepsy risk……………………………………………………………………………………………...…………………………………13

Figure S5. The causal effect of BE on generalized epilepsy risk …………………………………………………………………………………………………..………………14

Figure S6. The causal effect of BE on focal epilepsy risk………………………………………………………………………………………..…………………………………15

Figure S7. The causal effect of generalized epilepsy on GRED………………………………………………………………………………...…………………..………………16

Figure S8. The causal effect of generalized epilepsy on BE…………………………………………………………………………………...……………………………………17

Figure S9. Outliers identified by MR Radial…………………………………………………………………………………………………………..……………………………18

**Supplementary Tables**

**Table S1.** Detailed information of the studies and datasets used for MR analysis

| **Phenotype** | **Sample size** | **Population** | **Consortium** | **Year** | **Journal** | **References** |
| --- | --- | --- | --- | --- | --- | --- |
| Esophageal disease  Gastroesophageal reflux disease  Barrett’s esophagus  Epilepsy  Epilpsy  Generalized epilepsy  Focal epilepsy | 129,080/473,524  43,071/56,429  15,212/29,677  10,345/264,662  3,769/29,677  1,160/332,143  9,671/29,677  5,922/332,145 | European  European  ~86% European  European  ~83% European  European  ~85% European  European | -  -  ILAE  FinnGen  ILAE  FinnGen  ILAE  FinnGen | 2021  2021  2018  2022  2018  2022  2018  2022 | Gut  Gut  Nat Commun  -  Nat Commun  -  Nat Commun  - | Ong JS et al.  Ong JS et al.  Abou-Khalil et al.  -  Abou-Khalil et al.  -  Abou-Khalil et al.  - |

ILAE: International League Against Epilepsy; MR, mendelian randomization.

**Table S2.** Characteristics of selected SNPs associated with gastroesophageal reflux disease

| **SNP** | **Chr** | **Pos.** | **Effect allele** | **Other allele** | **EAF** | **Beta** | **SE** | ***P value*** | **R^2^** | **F statistic** |
| --- | --- | --- | --- | --- | --- | --- | --- | --- | --- | --- |
| rs569356  rs7541875  rs2782641  rs2815749  rs3766823  rs1937450  rs17379561  rs7527682  rs903678  rs6711584  rs13409451  rs4300861  rs12997558  rs6722661  rs1596747  rs7600261  rs1011407  rs6780459  rs2016933  rs7612999  rs2240326  rs7675588  rs7685686  rs2164300  rs1510719  rs10010963  rs1592757  rs11953061  rs329122  rs2744961  rs12204714  rs9396740  rs9372625  rs3828917  rs9373363  rs4713692  rs11762636  rs2043539  rs2396133  rs2106353  rs215614  rs903959  rs3863241  rs7032155  rs4382592  rs3793577  rs12357321  rs1021363  rs761777  rs10837002  rs2734839  rs7942368  rs773109  rs1479405  rs1716171  rs9542729  rs1334297  rs9529055  rs9517313  rs942065  rs10133111  rs9940128  rs12598916  rs7206608  rs12453010  rs7241572  rs1431196  rs12967855  rs9636202  rs2023878  rs1883842  rs2834005  rs2838771  rs9615905 | 1  1  1  1  1  1  1  1  1  2  2  2  2  2  2  2  2  3  3  3  3  4  4  4  4  4  5  5  5  6  6  6  6  6  6  6  7  7  7  7  7  8  8  9  9  9  10  10  10  11  11  11  12  12  12  13  13  13  13  14  14  16  16  16  17  18  18  18  19  19  20  21  21  22 | 29136686  190957589  44013355  72814783  32197257  66478840  98340139  189172684  201809918  104421692  144257639  22549441  41704580  100806588  193802478  212622818  60665768  104624105  65653157  35678337  50128386  80734978  3207142  67813017  140938116  159839313  103889998  120144025  133864599  34655000  152235339  17023108  98344031  31465917  143150043  33807638  2061111  12253880  109197067  126506598  32347335  142630782  73890335  122672771  134870755  23737627  21790476  106610839  134938075  38565727  113286490  76465362  56374695  15387519  123716376  31833578  58335375  66957533  99105892  94032065  103377321  53800754  60658751  82872628  50316131  77580712  50832102  35138245  18449238  18834124  41223062  34291708  46501576  48875699 | G  G  A  G  A  G  T  G  A  A  G  T  A  A  G  T  G  T  G  A  A  A  G  T  C  T  C  T  A  T  T  A  A  T  G  T  A  A  G  T  A  A  T  A  G  G  A  G  G  G  T  T  A  T  T  G  A  A  C  A  A  A  G  G  T  A  G  G  A  T  G  C  C  T | A  A  G  A  G  T  A  A  G  G  A  C  G  G  A  C  A  A  C  G  G  C  A  C  T  C  G  C  G  C  C  G  G  G  A  C  C  G  A  G  G  T  C  C  T  A  G  A  A  C  C  C  G  C  C  C  G  G  G  G  G  G  C  C  C  G  A  A  G  C  T  T  G  C | 0.1408  0.4261  0.6127  0.8010  0.1715  0.5377  0.1444  0.5373  0.3394  0.4520  0.3924  0.3821  0.3588  0.3647  0.4941  0.3064  0.1216  0.7466  0.7301  0.2453  0.4738  0.7946  0.4224  0.5233  0.3834  0.6164  0.3558  0.3389  0.4196  0.3584  0.6322  0.2488  0.3830  0.0418  0.2536  0.3678  0.1803  0.4187  0.4753  0.2315  0.6297  0.3993  0.5270  0.5919  0.6995  0.5383  0.3111  0.6420  0.2540  0.3512  0.6067  0.2147  0.3353  0.3217  0.7900  0.2024  0.7342  0.4756  0.3832  0.6340  0.1630  0.4218  0.2748  0.3229  0.3948  0.2091  0.4284  0.6704  0.2666  0.1924  0.2793  0.3150  0.6467  0.4582 | -0.0379  0.0274  0.0271  0.0389  0.0394  0.0316  0.0531  -0.0267  0.0277  0.0323  -0.0277  0.0307  0.0278  -0.0323  0.0311  0.0338  -0.0421  0.0306  -0.0310  0.0305  -0.0472  -0.0335  -0.0279  -0.0265  -0.0389  -0.0270  0.0311  0.0282  -0.0290  0.0292  -0.0288  -0.0315  -0.0377  0.0671  -0.0327  -0.0276  -0.0515  0.0272  0.0294  0.0367  -0.0329  0.0292  0.0325  0.0278  -0.0303  0.0270  0.0317  -0.0312  0.0345  0.0276  -0.0283  -0.0340  -0.0381  0.0315  0.0384  -0.0363  -0.0388  0.0267  0.0331  0.0307  0.0418  0.0333  -0.0333  0.0292  0.0297  0.0366  0.0324  -0.0365  -0.0350  -0.0363  0.0308  0.0297  -0.0281  0.0276 | 0.0069  0.0048  0.0049  0.0060  0.0064  0.0048  0.0069  0.0048  0.0051  0.0048  0.0049  0.0049  0.0050  0.0050  0.0048  0.0052  0.0074  0.0055  0.0054  0.0056  0.0048  0.0060  0.0049  0.0048  0.0049  0.0049  0.0050  0.0051  0.0049  0.0050  0.0050  0.0056  0.0050  0.0120  0.0056  0.0050  0.0063  0.0049  0.0048  0.0057  0.0050  0.0049  0.0048  0.0049  0.0053  0.0048  0.0052  0.0050  0.0055  0.0050  0.0049  0.0059  0.0051  0.0052  0.0059  0.0060  0.0055  0.0048  0.0049  0.0050  0.0065  0.0049  0.0054  0.0051  0.0049  0.0060  0.0049  0.0051  0.0055  0.0061  0.0054  0.0052  0.0051  0.0048 | 4.07E-08  1.61E-08  4.33E-08  1.07E-10  7.09E-10  7.07E-11  1.08E-14  3.13E-08  4.89E-08  2.66E-11  1.93E-08  5.43E-10  3.04E-08  1.15E-10  1.00E-10  9.47E-11  1.09E-08  3.14E-08  1.04E-08  4.90E-08  1.13E-22  1.80E-08  1.14E-08  4.13E-08  3.84E-15  4.92E-08  6.00E-10  3.10E-08  3.05E-09  5.81E-09  7.92E-09  1.47E-08  2.62E-14  2.27E-08  4.13E-09  3.07E-08  1.88E-16  2.24E-08  1.11E-09  1.37E-10  4.08E-11  2.99E-09  1.49E-11  1.63E-08  8.20E-09  2.49E-08  1.33E-09  5.10E-10  4.71E-10  4.03E-08  8.79E-09  9.54E-09  8.71E-14  9.85E-10  7.83E-11  1.41E-09  1.14E-12  3.11E-08  2.05E-11  8.45E-10  1.35E-10  8.06E-12  6.87E-10  1.46E-08  1.75E-09  9.49E-10  2.65E-11  1.09E-12  1.51E-10  3.04E-09  9.27E-09  9.42E-09  2.91E-08  1.21E-08 | 0.0004  0.0004  0.0004  0.0005  0.0004  0.0005  0.0007  0.0004  0.0004  0.0005  0.0004  0.0005  0.0004  0.0005  0.0005  0.0005  0.0004  0.0004  0.0004  0.0003  0.0011  0.0004  0.0004  0.0004  0.0007  0.0003  0.0004  0.0004  0.0004  0.0004  0.0004  0.0004  0.0007  0.0004  0.0004  0.0004  0.0008  0.0004  0.0004  0.0005  0.0005  0.0004  0.0005  0.0004  0.0004  0.0004  0.0004  0.0005  0.0005  0.0004  0.0004  0.0004  0.0007  0.0004  0.0005  0.0004  0.0006  0.0004  0.0005  0.0004  0.0005  0.0005  0.0004  0.0004  0.0004  0.0004  0.0005  0.0006  0.0005  0.0004  0.0004  0.0004  0.0004  0.0004 | 209.755  221.295  209.933  290.521  265.369  299.008  419.662  213.422  207.976  310.733  220.689  268.529  214.634  290.632  291.277  292.808  227.883  212.876  228.713  207.962  669.242  221.103  229.331  210.807  431.116  207.506  267.379  214.201  246.147  236.412  232.799  223.485  405.657  217.620  243.810  213.750  472.433  217.186  259.110  289.663  303.483  245.951  317.456  224.274  232.169  218.941  259.925  270.060  272.498  210.015  231.189  234.523  389.273  260.801  294.919  256.793  354.211  213.725  312.534  264.339  287.253  325.150  265.831  224.058  254.061  266.398  310.350  355.855  289.553  246.629  230.699  229.473  217.478  227.434 |

Chr, chromosome; EAF, Effect allele frequency; Pos, position; SE, standard error; SNP, single-nucleotide polymorphism. The threshold was set at *P* < 5 × 10^-8^.

**Table S3.** Characteristics of selected SNPs associated with Barrett’s esophagus.

| **SNP** | **Chr** | **Pos.** | **Effect allele** | **Other allele** | **EAF** | **Beta** | **SE** | ***P value*** | **R^2^** | **F statistic** |
| --- | --- | --- | --- | --- | --- | --- | --- | --- | --- | --- |
| rs1868915  rs10207635  rs3072  rs2861695  rs2597301  rs7720419  rs10039754  rs622217  rs10104032  rs10982622  rs11792928  rs1247942  rs739414  rs7187365  rs8102046 | 2  2  2  2  3  5  5  6  8  9  9  12  16  16  19 | 200015561  56040035  20878406  67846727  70909494  589343  28986950  160766770  9616664  100304531  129401550  114673723  73097956  86511915  18795578 | A  T  C  G  G  T  G  C  A  G  T  C  T  C  G | C  A  T  A  C  A  A  T  C  A  C  G  C  T  T | 0.5866  0.1345  0.3675  0.8061  0.6886  0.3788  0.5501  0.4818  0.3749  0.4617  0.2948  0.4054  0.7419  0.8230  0.5560 | 0.0893  0.1367  0.1104  -0.1072  -0.1140  0.0904  0.0838  -0.0911  0.0926  -0.0855  0.0977  -0.0961  -0.1011  -0.1114  -0.0917 | 0.0153  0.0220  0.0157  0.0188  0.0162  0.0155  0.0151  0.0150  0.0156  0.0152  0.0166  0.0154  0.0176  0.0195  0.0151 | 5.86E-09  5.19E-10  2.31E-12  1.28E-08  1.92E-12  5.72E-09  2.59E-08  1.37E-09  2.63E-09  1.76E-08  4.16E-09  3.81E-10  9.74E-09  1.20E-08  1.31E-09 | 0.0039  0.0044  0.0057  0.0036  0.0056  0.0038  0.0035  0.0041  0.0040  0.0036  0.0040  0.0045  0.0039  0.0036  0.0042 | 218.916  246.649  321.312  203.387  316.108  218.050  196.814  234.961  227.573  205.742  224.654  252.484  221.877  204.683  235.392 |

Chr, chromosome; EAF, Effect allele frequency; Pos, position; SE, standard error; SNP, single-nucleotide polymorphism. The threshold was set at *P* < 5 × 10^-8^.

**Table S4.** Characteristics of selected SNPs associated with epilepsy and its subtypes.

| **SNP** | **Trait** | **Chr** | **Pos.** | **Effect allele** | **Other allele** | **EAF** | **Beta** | **SE** | ***P value*** | **R^2^** | **F statistic** |
| --- | --- | --- | --- | --- | --- | --- | --- | --- | --- | --- | --- |
| rs6432877 | epilepsy | 2 | 166998767 | G | C | 0.255 | 0.0634 | 0.0086 | 1.70E-13 | 0.0015 | 68.739 |
| rs4671319 | epilepsy | 2 | 57950346 | A | G | 0.5415 | -0.0419 | 0.0073 | 8.07E-09 | 0.0009 | 39.123 |
| rs4638568 | epilepsy | 16 | 50045839 | A | G | 0.0605 | -0.0861 | 0.0157 | 4.00E-08 | 0.0008 | 37.861 |
| rs2212656 | focal epilepsy | 2 | 167000843 | A | C | 0.2504 | 0.0587 | 0.0102 | 7.30E-09 | 0.0013 | 51.005 |
| rs11890028 | generalized epilepsy | 2 | 166943277 | G | T | 0.2789 | -0.0757 | 0.0138 | 4.68E-08 | 0.0023 | 77.182 |
| rs887696 | generalized epilepsy | 2 | 191583507 | T | C | 0.6662 | -0.0727 | 0.0131 | 3.00E-08 | 0.0023 | 78.759 |
| rs4665630 | generalized epilepsy | 2 | 23898317 | T | C | 0.8886 | -0.1090 | 0.0199 | 4.30E-08 | 0.0024 | 78.834 |
| rs1402398 | generalized epilepsy | 2 | 58042241 | A | G | 0.6247 | -0.0866 | 0.0128 | 1.16E-11 | 0.0035 | 118.073 |
| rs1044352 | generalized epilepsy | 4 | 31147874 | T | G | 0.4213 | 0.0752 | 0.0126 | 2.17E-09 | 0.0028 | 92.417 |
| rs11943905 | generalized epilepsy | 4 | 46397617 | T | C | 0.2736 | 0.0775 | 0.0141 | 3.90E-08 | 0.0024 | 80.125 |
| rs10060382 | generalized epilepsy | 5 | 114401519 | T | C | 0.4968 | -0.0725 | 0.0126 | 7.50E-09 | 0.0026 | 88.189 |
| rs13200150 | generalized epilepsy | 6 | 128309768 | G | A | 0.3076 | -0.0782 | 0.0134 | 5.92E-09 | 0.0026 | 87.290 |
| rs68082256 | generalized epilepsy | 6 | 16971575 | A | G | 0.2059 | -0.0934 | 0.0155 | 1.70E-09 | 0.0029 | 95.687 |
| rs4794333 | generalized epilepsy | 17 | 46045495 | C | T | 0.3943 | -0.0736 | 0.0127 | 6.81E-09 | 0.0026 | 86.738 |
| rs2833098 | generalized epilepsy | 21 | 32183996 | A | G | 0.6299 | 0.0731 | 0.0130 | 1.70E-08 | 0.0025 | 83.460 |

Chr, chromosome; EAF, Effect allele frequency; Pos, position; SE, standard error; SNP, single nucleotide polymorphism. The threshold was set at *P* < 5 × 10^-8^.

**Table S5.** Forward causal relationships of two esophageal disease with epilepsy performed by MR before removing the outliers.

| **Exposure** | **nSNPs** | **Method** | **OR (95%CI)** | ***P value*** | **Q pval** | **intercept**  ***p* value** | **Global *P*** |
| --- | --- | --- | --- | --- | --- | --- | --- |
| GERD versus epilepsy | 74 | IVW | 1.078 (1.014, 1.146) | **0.016** | 0.127 |  |  |
|  |  | MR Egger | 0.778 (0.538, 1.125) | 0.186 |  | 0.083 |  |
|  |  | MR-PRESSO | 1.078 (1014., 1.146) | **0.018** |  |  | 0.129 |
|  |  | WM | 1.057 (0.972, 1.149) | 0.198 |  |  |  |
| BE versus epilepsy | 15 | IVW | 1.029 (0.977, 1.084) | 0.277 | 0.069 |  |  |
|  |  | MR Egger | 0.833 (0.946, 1.702) | 0.833 |  | 0.891 |  |
|  |  | MR-PRESSO | 1.029 (0.977, 1.083) | 0.295 |  |  | 0.074 |
|  |  | WM | 1.007 (0.946, 1.072) | 0.716 |  |  |  |
| GERD versus generalized epilepsy | 74 | IVW | 1.133 (0.977, 1.313) | 0.098 | 0.127 |  |  |
|  |  | MR Egger | 0.741 (0.300, 1.831) | 0.519 |  | 0.355 |  |
|  |  | **MR-PRESSO** | 1.133 (0.997, 1.313) | 0.102 |  |  | **1e-04** |
|  |  | WM | 1.144 (0.985, 1.329) | 0.079 |  |  |  |
| BE versus generalized epilepsy | 15 | IVW | 1.034 (0.964, 1.110) | 0.350 | 0.407 |  |  |
|  |  | MR Egger | 1.228 (0.671, 2.247) | 0.517 |  | 0.584 |  |
|  |  | MR-PRESSO | 1.034 (0.999, 1.071) | 0.366 |  |  | 0.403 |
|  |  | WM | 1.061 (0.960, 1.110) | 0.246 |  |  |  |
| GERD versus focal epilepsy | 74 | IVW | 1.059 (0.992, 1.131) | 0.084 | 0.701 |  |  |
|  |  | MR Egger | 0.804 (0.539, 1.200) | 0.289 |  | 0.175 |  |
|  |  | **MR-PRESSO** | 1.059 (0.995, 1.127) | 0.073 |  |  | 0.704 |
|  |  | WM | 1.022 (0.930, 1.122) | 0.653 |  |  |  |
| BE versus focal epilepsy | 15 | IVW | 1.021 (0.960, 1.086) | 0.506 | **0.049** |  |  |
|  |  | MR Egger | 1.003 (0.586, 1.716) | 0.992 |  | 0.947 |  |
|  |  | MR-PRESSO | 1.021 (0.960, 1.087) | 0.517 |  |  | 0.053 |
|  |  | WM | 1.036 (0.962, 1.116) | 0.343 |  |  |  |

BE, Barrett’s esophageal disease; CI, confidence interval; GERD, gastroesophageal reflux disease; IVW, inverse-variance weighted; MR, Mendelian randomization; MR-PRESSO, Pleiotropy Residual Sum and Outlier; nSNPs, number of single nucleotide polymorphisms; OR, odds ratio; SVS, small-vessel; Q_pval, *P*-value of the Cochran Q statistic; WM, weighted median.

**Table S6.** Reverse causal relationships of epilepsy with two esophageal disease performed by MR before removing the outliers.

| **Exposure** | **nSNPs** | **Method** | **OR (95%CI)** | ***P value*** | **Q pval** | **intercept**  ***p* value** | **Global *P*** |
| --- | --- | --- | --- | --- | --- | --- | --- |
| Epilepsy | 3 | IVW | 1.030 (0.805, 1.318) | 0.813 | **0.011** |  |  |
|  |  | MR Egger | 0.589 (0.306, 1.132) | 0.358 |  | 0.332 |  |
|  |  | WM | 1.093 (0.936, 1.277) | 0.260 |  |  |  |
| Generalized epilepsy | 8 | IVW | 1.010 (0.940, 1.085) | 0.789 | **0.016** |  |  |
|  |  | MR Egger | 1.314 (0.356, 4.856) | 0.696 |  | 0.062 |  |
|  |  | MR-PRESSO | 1.010 (0.940, 1.085) | 0.797 |  |  | **0.021** |
|  |  | WM | 1.053 (0.972, 1.105) | 0.290 |  |  |  |
| Focal epilepsy | 1 | Wald ratio | 1.107 (0.919, 1.332) | 0.285 |  |  |  |
| Epilepsy | 3 | IVW | 1.066 (0.632, 1.800) | 0.811 | 0.126 |  |  |
|  |  | MR Egger | 0.422 (0.054, 3.311) | 0.562 |  | 0.528 |  |
|  |  | WM | 1.338 (0.842, 2.126) | 0.218 |  |  |  |
| Generalized epilepsy | 9 | IVW | 1.051 (0.812, 1.360) | 0.079 | 0.240 |  |  |
|  |  | MR Egger | 5.044 (0.978, 26.000) | 0.094 |  | 0.119 |  |
|  |  | MR-PRESSO | 1.147 (0.985, 1.337) | 0.117 |  |  | 0.263 |
|  |  | WM | 1.063 (0.885, 1.277) | 0.510 |  |  |  |
| Focal epilepsy | 1 | Wald ratio | 1.395 (0.781, 2.492) | 0.261 |  |  |  |

CI, confidence interval; IVW, inverse-variance weighted; MR, Mendelian randomization; MR-PRESSO, Pleiotropy Residual Sum and Outlier; nSNPs, number of single nucleotide polymorphisms; OR, odds ratio; Q_pval, *P*-value of the Cochran Q statistic; WM, weighted median.

**Supplementary Figures**

**Figure S1.** The causal effect of GRED on epilepsy risk. (A) Scatter plot, (B) Funnel plot, (C) Forest plot, and (D) Leave one out plot. GERD, gastroesophageal reflux disease.

**Figure S2.** The causal effect of GRED on generalized epilepsy risk. (A) Scatter plot, (B) Funnel plot, (C) Forest plot, and (D) Leave one out plot. GERD, gastroesophageal reflux disease.


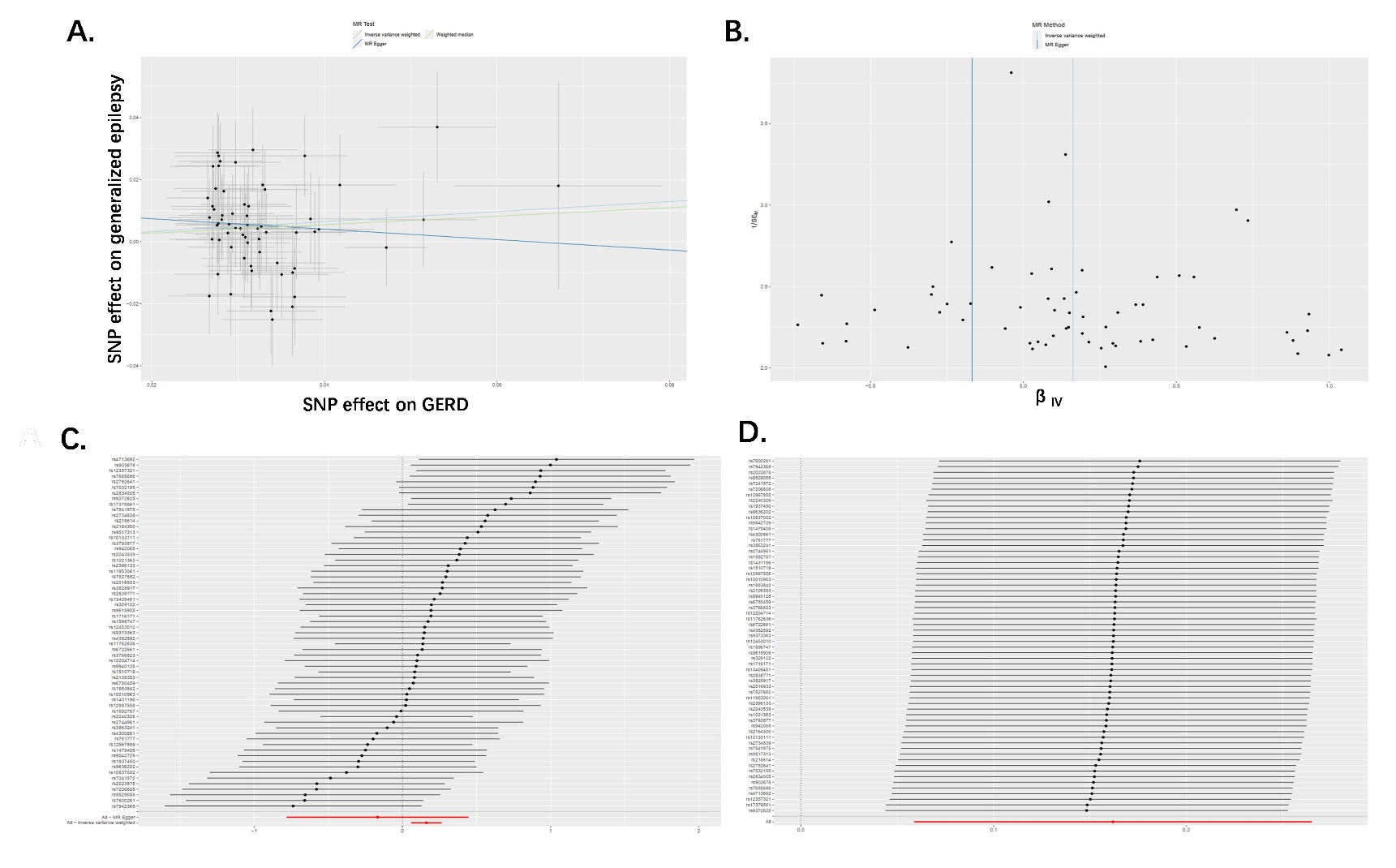


**Figure S3.** The causal effect of GRED on focal epilepsy risk. (A) Scatter plot, (B) Funnel plot, (C) Forest plot, and (D) Leave one out plot. GERD, gastroesophageal reflux disease.

**Figure S4.** The causal effect of BE on epilepsy risk. (A) Scatter plot, (B) Funnel plot, (C) Forest plot, and (D) Leave one out plot. BE, Barrett’s esophagus.

**Figure S5.** The causal effect of BE on generalized epilepsy risk. (A) Scatter plot, (B) Funnel plot, (C) Forest plot, and (D) Leave one out plot. BE, Barrett’s esophagus.

**Figure S6.** The causal effect of BE on focal epilepsy risk. (A) Scatter plot, (B) Funnel plot, (C) Forest plot, and (D) Leave one out plot. BE, Barrett’s esophagus.

**Figure S7.** The causal effect of generalized epilepsy on GRED. (A) Scatter plot, (B) Funnel plot, (C) Forest plot, and (D) Leave one out plot. GERD, gastroesophageal reflux disease.

**Figure S8.** The causal effect of generalized epilepsy on BE. (A) Scatter plot, (B) Funnel plot, (C) Forest plot, and (D) Leave one out plot. BE, Barrett’s esophagus.

**Figure S9**. Outliers identified by MR Radial.


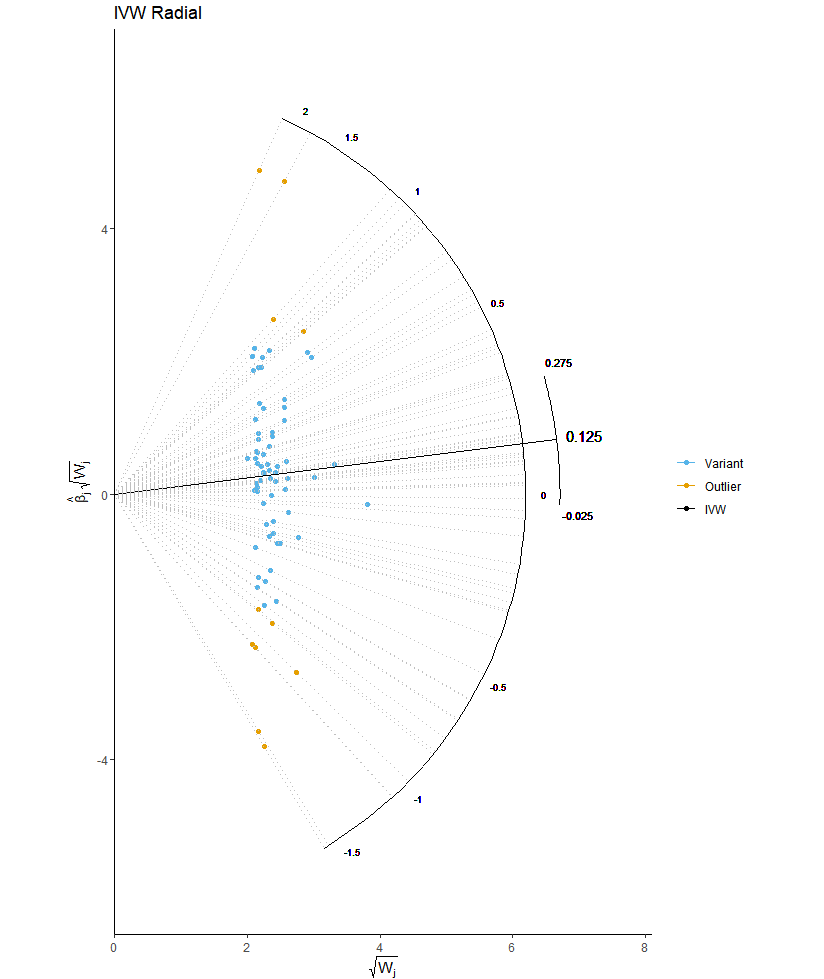

Supplement: Supplementary file 1 — Table S1. Detailed information of the studies and datasets used for MR analysis Table S2. Characteristics of selected SNPs associated with gastroesophageal reflux disease Table S3. Characteristics of selected SNPs associated with Barrett's esophagus. Table S4. Characteristics of selected SNPs associated with epilepsy and its subtypes. Table S5. Forward causal relationships of two esophageal disease with epilepsy performed by MR before removing the outliers. Table S6. Reverse causal relationships of epilepsy with two esophageal disease performed by MR before removing the outliers. Figure S1. The causal effect of GRED on epilepsy risk. (A) Scatter plot, (B) Funnel plot, (C) Forest plot, and (D) Leave one out plot. GERD, gastroesophageal reflux disease. Figure S2. The causal effect of GRED on generalized epilepsy risk. (A) Scatter plot, (B) Funnel plot, (C) Forest plot, and (D) Leave one out plot. GERD, gastroesophageal reflux disease. Figure S3. The causal effect of GRED on focal epilepsy risk. (A) Scatter plot, (B) Funnel plot, (C) Forest plot, and (D) Leave one out plot. GERD, gastroesophageal reflux disease. Figure S4. The causal effect of BE on epilepsy risk. (A) Scatter plot, (B) Funnel plot, (C) Forest plot, and (D) Leave one out plot. BE, Barrett's esophagus. Figure S5. The causal effect of BE on generalized epilepsy risk. (A) Scatter plot, (B) Funnel plot, (C) Forest plot, and (D) Leave one out plot. BE, Barrett's esophagus. Figure S6. The causal effect of BE on focal epilepsy risk. (A) Scatter plot, (B) Funnel plot, (C) Forest plot, and (D) Leave one out plot. BE, Barrett's esophagus. Figure S7. The causal effect of generalized epilepsy on GRED. (A) Scatter plot, (B) Funnel plot, (C) Forest plot, and (D) Leave one out plot. GERD, gastroesophageal reflux disease. Figure S8. The causal effect of generalized epilepsy on BE. (A) Scatter plot, (B) Funnel plot, (C) Forest plot, and (D) Leave one out plot. BE, Barrett's esophagus. Figure S9. Outliers identified by M [file BRB3-13-e3117-s001.docx]
